# Hierarchical affective predictive imbalance in adolescent borderline personality disorder: task-evoked fMRI evidence from multimodal machine learning

**DOI:** 10.3389/fnhum.2026.1875156

**Published:** 2026-10-15

**Authors:** Mingzhao Ma, Xueying Wang, Zeming Tan, Jun Liu, Qian Xiao, Ting Hu, Alessandro Grecucci, Xiaoping Yi

**Affiliations:** 1 Department of Radiology, The Second Xiangya Hospital of Central South University, Central South University, Changsha, Hunan, China; 2 Department of Nuclear Medicine, Chongqing University Three Gorges Hospital, Chongqing University, Chongqing, China; 3 Department of Radiology, Chongqing University Three Gorges Hospital, Chongqing University, Chongqing, China; 4 Department of Neurosurgery, Xiangya Hospital, Central South University, Changsha, Hunan, China; 5 Mental Health Center of Xiangya Hospital, Central South University, Changsha, Hunan, China; 6 School of Computer Science and Engineering, Central South University, Changsha, Hunan, China; 7 Department of Education, Psychology and Communication Sciences, University of Bari, Bari, Italy; 8 Medical Imaging Center (MIC), Clinical Research Center (CRC), Medical Pathology Center (MPC), Cancer Early Detection and Treatment Center (CEDTC) and Translational Medicine Research Center (TMRC), Chongqing University Three Gorges Hospital, Chongqing University, Chongqing, China; 9 School of Medicine, Chongqing University, Chongqing, China; 10 FuRong Laboratory, Changsha, Hunan, China; 11 National Engineering Research Center of Personalized Diagnostic and Therapeutic Technology, Xiangya Hospital, Changsha, Hunan, China

**Keywords:** adolescent BPD, affective inhibitory control, multimodal machine learning, neuroimaging biomarkers, predictive processing, task-fMRI

## Abstract

**Background:**

Predictive-processing accounts of BPD, including the hierarchical-prior-imbalance account proposed by Herzog, offer a framework for interpreting affective instability and impulsivity. The present task-fMRI study evaluates whether discriminative task-evoked patterns are consistent with this framework; because it does not directly estimate prediction errors, precision weighting, or hierarchical belief updating, it is not a mechanistic test of predictive processing.

**Methods:**

To evaluate this question, this study developed a leakage-controlled multimodal machine learning framework for individualized classification of adolescents with BPD and healthy controls based on emotional Go/No-Go task-fMRI activity. Three feature domains were constructed: functional connectivity (FC), task-evoked general linear model (GLM)-derived temporal features, and clinical variables. Seven feature settings, including single-modality, dual-modality, and full multimodal combinations, were evaluated using multiple conventional machine learning classifiers, including logistic regression, support vector machine, k-nearest neighbors, Gaussian naive Bayes, multilayer perceptron, random forest, and XGBoost or gradient boosting.

**Results:**

The results showed that task-evoked GLM-derived features provided the strongest single-modality discriminative information (AUC of 0.881 ± 0.055). SHAP analysis confirmed the predominance of GLM-derived features, accounting for 83.1% of total model attribution, compared to 9.5 and 7.5% for functional connectivity and clinical variables, respectively. At the neural level, discriminative features were distributed across visual/perceptual regions (calcarine, cuneus, occipital cortex), temporoparietal/default-mode hubs (angular and supramarginal gyri), frontal control regions (inferior frontal gyrus), and subcortical structures (putamen). This distributed pattern aligns with a hierarchical organization spanning perceptual input, contextual/self-referential processing, and top-down inhibitory control systems, consistent with the proposed model of hierarchical affective predictive imbalance.

**Conclusion:**

Task-evoked GLM-derived features were the primary source of discriminative information for adolescent BPD, with functional connectivity and clinical variables providing modest additional value. The resulting classification pattern is consistent with hierarchical predictive-processing accounts but does not establish a specific predictive-processing mechanism. The same regional pattern may also reflect visual attention, salience processing, and executive inhibitory control.

## 1 Introduction

Borderline personality disorder (BPD) is a severe and heterogeneous psychiatric condition characterized by affective instability, impulsivity, disturbed interpersonal functioning, and difficulties in self-regulation. Although BPD has traditionally been studied in adults, increasing evidence suggests that clinically significant borderline features can emerge during adolescence, a developmental stage marked by rapid neurobiological, emotional, and social changes. Early identification of adolescent BPD is clinically important because persistent emotional dysregulation, self-harm risk, interpersonal instability, and functional impairment may lead to long-term adverse outcomes if not recognized and treated in time. Beyond its clinical heterogeneity, a central challenge in adolescent BPD lies in its diagnostic and mechanistic ambiguity. Symptoms frequently overlap with mood, anxiety, and trauma-related conditions, as well as with normative developmental variability, limiting the specificity of categorical diagnosis. These challenges have motivated a shift toward neurobiologically grounded and data-driven approaches aimed at identifying objective markers of individual-level vulnerability. However, such approaches require a coherent theoretical framework linking distributed brain dysfunction to the core features of borderline psychopathology (American Psychiatric Association, 2022; Leichsenring et al., 2011; Gunderson et al., 2018; Kaess et al., 2014; Sharp and Fonagy, 2015; Guilé et al., 2018; Chanen et al., 2020; Crowell et al., 2009).

Functional magnetic resonance imaging (fMRI) provides a non-invasive approach for investigating neural alterations associated with psychiatric symptoms. In particular, task-based fMRI enables the examination of brain responses under controlled cognitive and emotional demands, offering a unique opportunity to probe the dynamic regulation of affect and action. Emotional Go/No-Go paradigms are especially relevant for adolescent BPD, as they require the inhibition of prepotent responses under affective interference, directly engaging processes related to impulsivity, emotion regulation, and salience attribution. Crucially, such paradigms can be interpreted within a predictive control framework, in which behavior depends on the ability to generate, update, and regulate predictions about emotionally salient stimuli. From this perspective, task-evoked neural responses provide a more direct window into affective predictive regulation than resting-state activity, which primarily reflects intrinsic or baseline network organization. While resting-state measures have been informative in identifying latent network-level alterations, they do not directly test how these systems operate when predictive regulation must be actively engaged. Accordingly, task-evoked fMRI may capture disease-relevant neural dynamics that are more proximal to symptom expression. Nevertheless, how best to transform task-fMRI signals into reliable individual-level biomarkers remains an open methodological question (Hare et al., 2008; Tottenham et al., 2011; Brown et al., 2015; Logothetis, 2008; Friston et al., 1994; Worsley and Friston, 1995; Lindquist, 2008; Liu et al., 2025).

While resting-state approaches have provided important insights into intrinsic network organization, they do not directly capture how these systems operate under affective demands (Grecucci et al., 2025). Task-based paradigms offer a critical advantage in this context, as they allow direct assessment of how predictive control mechanisms are engaged during emotionally salient conditions, which may represent the level at which dysfunction in adolescent BPD is most strongly expressed.

Traditional neuroimaging studies of BPD have mainly focused on group-level statistical differences, such as abnormal activation in prefrontal, limbic, temporal, and occipital regions. Although these studies have improved our understanding of the neurobiological basis of BPD (Xiao et al., 2024), group-level findings do not directly provide individualized diagnostic predictions. Supervised machine learning provides a complementary framework to address these limitations by identifying multivariate patterns that discriminate patients from healthy controls at the individual level. Critically, such approaches are well suited to test models of distributed hierarchical dysfunction, as they can integrate high-dimensional features spanning multiple brain systems into a single predictive representation. In this context, machine learning can be used not only as a classification tool, but as a means to probe whether hypothesized large-scale architectures, such as hierarchical affective predictive imbalance, are expressed as stable, generalizable patterns in the data (Krause-Utz et al., 2014; Schulze et al., 2016; Ruocco et al., 2013; Ruocco et al., 2012; Degasperi et al., 2021; Silbersweig et al., 2007; Minzenberg et al., 2007; Herpertz et al., 2001; Donegan et al., 2003; van Zutphen et al., 2015; Orrù et al., 2012; Arbabshirani et al., 2017; Vieira et al., 2017; Woo et al., 2017; Varoquaux, 2018; Scheinost et al., 2019; Poldrack et al., 2020; Eklund et al., 2016; Nichols and Holmes, 2002).

Several types of features can be derived from fMRI data for machine learning-based psychiatric classification. Functional connectivity (FC) represents inter-regional synchronization and can reflect large-scale network organization. In contrast, task-evoked general linear model (GLM) features characterize condition-specific neural responses and thus capture the dynamic engagement of brain systems under cognitive and emotional demands. Clinical variables, including behavioral and psychological measures, may further contribute complementary phenotypic information. Within a hierarchical predictive framework, these feature domains can be interpreted as probing distinct levels of system expression. FC may reflect the baseline organization of the system (i.e., latent architecture), whereas task-evoked GLM features capture its functional expression during active predictive regulation. From this perspective, if adolescent BPD is characterized by a hierarchical affective predictive imbalance, the most informative signal should emerge when the system is engaged under affective control demands, rather than in its intrinsic resting configuration. In particular, it remains unclear whether multimodal integration substantially improves classification performance or whether task-evoked activation features alone already contain the dominant discriminative signal (Biswal et al., 1995; Rubinov and Sporns, 2010; Smith, 2012; Power et al., 2012; Satterthwaite et al., 2012; Tzourio-Mazoyer et al., 2002; Woo et al., 2017; Pedregosa et al., 2011; Abraham et al., 2014).

To address these issues, the present study evaluated whether multimodal task-fMRI classification patterns in adolescent BPD are consistent with a hierarchical affective predictive imbalance (HAPI) account. Predictive-processing explanations of BPD are not new: Herzog et al. proposed that imbalanced hierarchical priors may contribute to affective instability and impulsivity (Herzog et al., 2022). Task-fMRI machine-learning classification has also previously been examined in BPD during emotion regulation (Cremers et al., 2021), and structural MRI classification has achieved an AUC of 0.88 (Grecucci et al., 2022). Accordingly, the incremental contribution of the present study is the leakage-controlled comparison of functional connectivity, task-evoked dynamic descriptors, and clinical variables in a relatively large adolescent sample, rather than the first neuroimaging machine-learning classification of BPD. The study does not directly estimate prediction errors or precision weighting and therefore treats HAPI as an interpretive framework rather than a proven mechanism.

Our multimodal integration strategy--concatenating functional connectivity, task-evoked activation, and phenotypic data--is consistent with broader multimodal fusion approaches increasingly used in medical imaging to integrate complementary sources of information (Zhang et al., 2025). By integrating these domains, we move beyond univariate analyses to examine whether the proposed predictive imbalance is expressed as a distributed, generalizable pattern across neural and clinical feature spaces (Yang et al., 2026).

The contributions of this study are threefold. First, we constructed a multimodal task-fMRI-based machine learning framework that integrates FC, GLM-derived activation features, and clinical variables for adolescent BPD classification. Second, we systematically compared single-modality, pairwise-modality, and full multimodal feature settings across multiple machine learning classifiers, providing a comprehensive benchmark rather than relying on a single model. Third, we adopted a leakage-controlled cross-validation strategy in which task-evoked regions and all preprocessing steps were determined within the training folds, thereby improving the reliability of the reported classification performance (Varoquaux, 2018; Scheinost et al., 2019; Poldrack et al., 2020; Eklund et al., 2016; Nichols and Holmes, 2002).

By combining a hypothesis-driven interpretive framework with systematic benchmarking, this study examines whether leakage-controlled multimodal classification patterns are consistent with HAPI while providing an interpretable approach for individualized adolescent BPD assessment.

## 2 Materials and methods

### 2.1 Study design and overall workflow

This study was designed as a hypothesis-driven evaluation of whether the observed multimodal classification patterns are consistent with the hierarchical affective predictive imbalance (HAPI) framework rather than as a purely exploratory machine learning pipeline. Within this framework, the three feature domains were mapped onto distinct levels of system expression. Functional connectivity features were used to index large-scale network organization; task-evoked GLM-derived features were used to capture the dynamic deployment of neural responses during affective inhibitory control; and clinical variables were used to represent the phenotypic expression of disorder-related dysfunction. By comparing the discriminative value of these feature domains under the same leakage-controlled cross-validation framework, the study aimed to determine whether adolescent BPD-related information is primarily expressed at the level of task-evoked predictive regulation, intrinsic network organization, or clinical phenotype.

In the input stage, task-fMRI data and clinical data were collected for each participant. In the feature construction stage, FC matrices were vectorized to represent inter-regional synchronization patterns, task-evoked GLM analysis was used to identify activation-related brain regions and extract regional temporal descriptors, and clinical variables were processed after excluding variables that could directly encode diagnostic grouping or disease-history information. These feature blocks were then organized into single-modality, dual-modality, and full multimodal feature settings (Friston et al., 1994; Tzourio-Mazoyer et al., 2002; Pedregosa et al., 2011; Abraham et al., 2014).

In the process stage, all models were evaluated using five-fold cross-validation. The same fold partitions were used across feature settings and classifiers to ensure a fair comparison. For each fold, feature preprocessing, normalization, feature selection, dimensionality reduction, and classifier training were performed using the training data only. The fitted transformations were then applied to the corresponding held-out test fold. This fold-specific design was used to reduce information leakage and to ensure that model performance reflected generalization to unseen participants (Varoquaux, 2018; Scheinost et al., 2019; Poldrack et al., 2020; Eklund et al., 2016; Nichols and Holmes, 2002).

Multiple conventional machine learning classifiers were evaluated, including logistic regression, support vector machine, k-nearest neighbors, Gaussian naive Bayes, multilayer perceptron, random forest, and XGBoost or gradient boosting-based models. The output stage included two types of results: classification performance metrics and model interpretation results. Classification performance was summarized using accuracy, AUC, F1 score, precision, recall/sensitivity, and specificity. Model interpretation was further conducted using SHAP analysis to quantify the relative contributions of different feature sources and identify the most influential task-evoked regional features (Breiman, 2001; Cortes and Vapnik, 1995; Cover and Hart, 1967; Friedman, 2001; Chen and Guestrin, 2016; Lundberg and Lee, 2017).

To improve reproducibility and computational efficiency, fold-specific feature matrices generated by the upstream preprocessing pipeline were saved and reused for the classifier comparison stage. This design ensured that all traditional machine learning classifiers were evaluated on identical fold-specific inputs and that computationally expensive GLM region construction did not need to be repeatedly performed during model benchmarking.

### 2.2 Participants

The source cohort available for the present study comprised 258 adolescents aged 12–17 years, including 129 individuals with borderline personality disorder (BPD) and 129 healthy controls (HC). Seven BPD participants were excluded because of missing data in one or more retained clinical scales. Therefore, the final multimodal task-fMRI dataset included 251 adolescents (122 BPD and 129 HC) with usable emotional Go/No-Go fMRI and complete retained variables. Participants were recruited from outpatient clinics at the Mental Health Center of Xiangya Hospital, Central South University, Changsha.

The BPD participants were drawn from the same parent clinical cohort and ethics protocol (IRB approval no. 2022020227) reported by Yi et al. (2026). Among the 129 adolescents with BPD in that cohort, 22.5% reported psychoactive medication use within the 2 months before MRI, including antidepressants, antipsychotics, and mood stabilizers. Medication was not discontinued for research purposes; the 24-h instruction referred to avoiding alcohol and other psychoactive substances before scanning and should not be interpreted as a medication washout. Seven BPD participants were excluded from the present analysis because of missing data in one or more retained clinical scales, leaving 122 BPD participants in the task-fMRI analytic sample.

Inclusion criteria for the adolescents with BPD included the following: (1) patients aged between 12 and 17 years fulfilling the diagnostic criteria for BPD in the Diagnostic and Statistical Manual of Mental Disorders, 4th Edition (DSM-IV) (21838207), having at least five out of nine criteria; (2) patients in whom symptoms had been stable for at least 2 years, and the symptoms could not be completely interpreted by the criteria for DSM-IV Axis I psychiatric disorders; and for whom the total score on the Borderline Personality Feature Scale for Children (BPFS) was greater than the demarcated score of 66; and (3) patients who were right-handed. For the HC group, inclusion criteria included the following: age between 12 and 17 years with intelligence quotient (IQ) > 80, and no history of psychiatric disorders or psychoactive medication. Exclusion criteria for both groups included the following: schizophrenia spectrum disorder, bipolar spectrum disorder, post-traumatic stress disorder, neurodevelopmental disorders, major depressive disorder, alcohol and/or drug dependence, neurological disorders, IQ ≤ 80, and left-handedness. All participants were required to avoid alcohol and psychoactive substances for at least 24 h before the brain MRI scan. *Balanced class weights were assigned during classifier training to account for the slight group imbalance*.

### 2.3 MRI acquisition and preprocessing

All brain MRI data were acquired on the same Siemens MAGNETOM Prisma 3 T MRI scanner. Both structural and functional brain MRI data were acquired for each participant. Structural imaging with a T1-weighted magnetization prepared–rapid gradient echo (MPRAGE) sequence for whole-brain coverage was acquired, with the following parameters: voxel size = 1.0 × 1.0 × 1.0 mm^3^, acquisition matrix = 256 × 256 mm^2^, repetition time = 2,300 ms, echo time = 2.03 ms, flip angle = 9°, and 176 slices with no gap. Functional magnetic resonance imaging (fMRI) data were acquired with an echo-planar imaging sequence, with the following parameters: echo time = 30 ms, repetition time = 2,000 ms, matrix size = 64 × 64, flip angle = 90°, number of time points = 250, field of view = 240 × 240 mm, slice thickness = 4 mm, gap = 0.4 mm, and 30 axial slices.

Spatial preprocessing of fMRI datasets was conducted using the SPM12 software.^1^ The fMRI images were corrected for temporal differences between slices and head movement. The fMRI images were realigned with reference to the first scan, spatially normalized using the SPM12 echo planar imaging template and resampled to an iso-voxel size of 3 mm × 3 mm × 3 mm. The images were smoothed by an 8 mm × 8 mm × 8 mm Gaussian kernel to reduce spatial noise.

### 2.4 Emotional go/no-go task design

During fMRI scanning, participants performed an emotional Go/No-Go task adapted from Wessa et al. (2007) (17403978), requiring response inhibition under affective interference. Prior to scanning, all participants completed a training session to ensure task comprehension. Participants were instructed to identify happy, sad, and neutral facial expressions from standardized facial affect pictures (Ekman and Friesen, 1976) and to press a button when a target face appeared (Go trials) while withholding their response when a distractor face appeared (No-Go trials).

Task structure: The task comprised six emotional Go/No-Go test blocks and two control Go/No-Go blocks. The six emotional blocks paired happy, sad, and neutral facial expressions (regardless of gender). The two control blocks used neutral male targets with female distractors and neutral female targets with male distractors. Each block lasted 36 s and contained 16 target (Go) trials and 8 distractor (No-Go) trials, appearing continuously in a pseudo-random sequence.

Stimulus timing: Each facial stimulus was presented for 500 ms, with an inter-stimulus interval of 1,000 ms. Before the start of each block, participants read a 4,000-ms instruction presented on the screen. Response time and accuracy were recorded using E-Prime software (Psychology Software Tools, Pittsburgh, PA).

Primary contrast: The main contrast of interest for the GLM analysis was happy No-Go versus neutral No-Go. In this contrast, participants were required to withhold their responses to both happy and neutral facial stimuli. The No-Go condition tested individuals’ ability to inhibit prepotent response tendencies, reflecting cognitive control ability. Happy faces were selected as a condition of primary interest because they represent real-life social and reward cues that are particularly salient for adolescents. Using happy faces as No-Go stimuli allowed us to examine the unique interference pattern of positive emotional arousal on response inhibition control, assessing whether adolescents with BPD were more prone to losing control or acting impulsively when confronted with positive emotional triggers. Behavioral performance indices, including omission errors, No-Go commission errors, and reaction-time-related measures across emotional conditions, were retained as phenotypic task-performance variables within the clinical feature modality. Participants with missing values in the retained clinical variables were excluded prior to model construction; therefore, no missing-value imputation was performed in the final analysis.

The emotional Go/No-Go paradigm was therefore selected because it simultaneously engages affective processing, inhibitory control, contextual prediction, and salience allocation, making it particularly suitable for evaluating task-evoked patterns within the HAPI interpretive framework.

### 2.5 Functional connectivity feature construction

Functional connectivity features were used to characterize inter-regional synchronization patterns across brain regions. For each participant, the FC representation was obtained from the corresponding functional connectivity matrix. The upper triangular elements of the connectivity matrix were vectorized to form a one-dimensional feature vector, thereby avoiding redundant symmetric entries (Biswal et al., 1995; Rubinov and Sporns, 2010; Smith, 2012; Power et al., 2012; Satterthwaite et al., 2012; Tzourio-Mazoyer et al., 2002).

Let $F_{i}$ denote the functional connectivity matrix of participant i. The FC feature vector was represented as FC vectorization in Equation (1):

$$x_{FC,i}=\to \left(upper\left(F_{i}\right)\right)$$ (1)

The fold-specific FC features were used consistently across all classifiers. Any preprocessing, feature reduction, or normalization associated with the FC block was fitted using the training data only and then applied to the corresponding held-out test samples (Varoquaux, 2018; Scheinost et al., 2019; Poldrack et al., 2020; Eklund et al., 2016; Nichols and Holmes, 2002).

### 2.6 Task-evoked GLM-derived temporal feature construction

Task-evoked fMRI features were derived from GLM-based task activation analysis. For each participant, task-related neural responses during the emotional Go/No-Go paradigm were modeled using a general linear model, with task conditions entered as regressors and convolved with the canonical hemodynamic response function. Head-motion parameters were included as nuisance covariates. The primary task contrast was happy No-Go versus neutral No-Go, which was used to capture affective inhibitory control-related activation during emotional No-Go processing.

To avoid information leakage, GLM-derived task-relevant regions were identified separately within each cross-validation fold using training subjects only. First-level contrast maps were entered into a fold-specific second-level GLM, and significant clusters were identified using a predefined thresholding configuration (alpha = 0.05, cluster threshold = 10 voxels, two-sided testing, and false-positive-rate height control). The resulting fold-specific clusters were then applied to both training and held-out test participants for regional time-series extraction. Thus, test subjects did not contribute to region definition or feature selection. Because the selected clusters could vary across folds, stable regions recurring across folds were emphasized in the main interpretation, whereas fold-specific regions were treated as exploratory model-relevant features.

For each participant, voxel signals were averaged within each fold-specific GLM-derived region at every acquisition time point of the four-dimensional preprocessed, covariate-regressed BOLD run. Ten temporal statistics were then calculated from the resulting regional BOLD time course: mean, standard deviation, minimum, maximum, median, first-difference mean, first-difference standard deviation, 25th percentile, 75th percentile, and 90th percentile. These descriptors were not based on trial-wise beta series and were not calculated from a second-stage GLM residual time series. Statistics were concatenated across regions to form the final GLM-derived temporal feature vector. Dynamic statistics for region r of participant i in Equation (2):

$$\begin{array}{l} g_{i,r}=[mean,std,\min,\max,median,\\ \;\;\;\;\;\;\;\;\;\;\;\;\;\;diff_{mean},diff_{std},P25,P75,P90] \end{array}$$ (2)

Concatenation across R GLM-derived regions in Equation (3):

$$x_{GLM,i}=concat\left(g_{i,1},g_{i,2},\ldots ,g_{i,R}\right)$$ (3)

These temporal descriptors were intended to capture complementary aspects of task-evoked neural dynamics, including response amplitude, variability, and temporal fluctuation patterns, which are thought to reflect the moment-to-moment engagement and stability of neural systems underlying affective predictive control, rather than representing arbitrary summary statistics. The GLM-derived dynamic features were standardized within each training fold. The scaler fitted on the training data was applied to the corresponding test data. No test-label information was used during standardization or feature construction.

### 2.7 Clinical feature processing

Clinical variables were incorporated as an additional phenotypic modality. The clinical feature block included 38 variables covering demographic, symptom-level, cognitive, and task-behavioral measures. Participants with missing values in the retained clinical variables were manually excluded before model construction. Therefore, the final matched multimodal dataset included 251 participants with complete clinical data across all 38 retained clinical variables, and no clinical-value imputation was performed in the final analysis. Specifically, these variables included years of education; emotional dysregulation and borderline symptom measures; depressive, anxiety, trauma-related, impulsivity, self-harm/suicidal ideation, sleep, manic, and global functioning scores; as well as cognitive and behavioral indices from Stroop, Trail Making, digit span, memory reproduction, and emotional Go/No-Go task performance (American Psychiatric Association, 2022; Leichsenring et al., 2011; Gunderson et al., 2018; Kaess et al., 2014; Sharp and Fonagy, 2015; Guilé et al., 2018; Chanen et al., 2020; Crowell et al., 2009).

Before model training, variables that could directly encode diagnostic grouping or disease-history information were excluded. These included diagnostic group indicators, disease duration, and age at onset. The purpose of this filtering step was to prevent the classifier from relying on shortcut variables that trivially reflected diagnostic status rather than generalizable phenotypic patterns.

Clinical features were processed within each cross-validation fold using the same training-only principle as the imaging features. Continuous clinical features were standardized using the training-fold mean and standard deviation, and the fitted scaler was applied to the held-out fold. Unlike the very high-dimensional FC matrix, the 38 retained clinical variables were not subjected to ANOVA-PCA because they formed a comparatively small, clinically interpretable feature set after shortcut variables were removed. This asymmetric choice may nevertheless increase estimation variance and partly contribute to weaker clinical-only performance; therefore, lower clinical-model performance should not be interpreted as absence of predictive clinical information.

### 2.8 Multimodal feature settings

To evaluate the independent and complementary value of each modality, seven feature settings were constructed: FC only, GLM only, Clinical only, FC + GLM, FC + Clinical, GLM + Clinical, and FC + GLM + Clinical. For combined settings, the corresponding feature blocks were concatenated along the feature dimension (Tables 1). Full multimodal feature representation in Equation (4):

$$X\_\mathrm{All}=\left[X\_\mathrm{FC, X}\_\mathrm{GLM, X}\_\mathrm{Clinical}\right]$$ (4)

This design allowed us to compare single-modality performance, dual-modality performance, and full multimodal performance under the same cross-validation and classifier evaluation framework. In addition, the dual-modality settings can be interpreted as leave-one-modality-out comparisons, enabling assessment of the relative contribution of each feature source.

### 2.9 Feature selection and dimensionality reduction

Feature selection and dimensionality reduction were performed within each cross-validation fold using training data only. For FC features, the upper triangular elements of each connectivity matrix were first vectorized and standardized within the training fold. Zero-variance features were removed using VarianceThreshold. Next, univariate feature selection was performed using SelectKBest with the ANOVA F-test (f_classif), retaining up to 256 FC features within each training fold. Principal component analysis was then applied to the selected FC features, retaining up to 32 components, with the exact number constrained by the training sample size and feature dimensionality. All fitted transformations, including standardization and preprocessing, variance filtering, feature selection, and PCA, were estimated using the training data only and then applied to the held-out test fold. GLM-derived regions were selected within each training fold through second-level GLM thresholding, and clinical features were not subjected to additional univariate feature selection after exclusion of diagnostic shortcut variables.

### 2.10 Machine learning classifiers

Multiple conventional classifiers were included as a robustness analysis of the predictive signal across model families with different inductive biases, rather than as an unconstrained search for the best-performing algorithm. Logistic regression was included as a linear baseline, SVM as a kernel-based classifier capable of modeling nonlinear decision boundaries, KNN as an instance-based method sensitive to local sample structure, Gaussian naive Bayes as a probabilistic baseline, MLP as a simple neural network model, and RF/XGBoost as ensemble-based classifiers capturing nonlinear feature interactions. If the HAPI-related predictive signal is robust, models using different assumptions should show a convergent pattern regarding the relative contribution of FC, GLM, and clinical features (Breiman, 2001; Cortes and Vapnik, 1995; Cover and Hart, 1967; Friedman, 2001; Chen and Guestrin, 2016; Lundberg and Lee, 2017). Predicted probability generated by classifier $f_{theta}$ for participant i in Equation (5):

$$p_{i}=f_{theta}\left(x_{i}\right)$$ (5)

The use of multiple classifiers was not intended to optimize performance per se, but to assess the stability and generalizability of the predictive signal across models with different inductive biases.

Hyperparameters were predefined based on prior neuroimaging machine-learning practice, small-sample considerations, and pilot implementation stability, rather than optimized using held-out test folds. No test-fold data were used for hyperparameter tuning, and the same settings were applied across feature settings and folds. This prespecification reduced optimization bias but did not constitute an exhaustive comparison of model families. Algorithms that are particularly sensitive to tuning, including XGBoost, may therefore have been disadvantaged; definitive algorithm ranking would require fully nested hyperparameter optimization in an independent or larger cohort (Tables 2).

### 2.11 Cross-validation and leakage control

Model performance was evaluated using five-fold cross-validation. In each fold, the model was trained on four-fifths of the data and tested on the remaining held-out fold. The same fold division was used for all feature settings and classifiers, allowing direct performance comparison (Varoquaux, 2018; Scheinost et al., 2019; Poldrack et al., 2020; Eklund et al., 2016; Nichols and Holmes, 2002).

To quantify feature importance across folds, SHAP values were computed for the fitted model using the corresponding held-out test fold. For each feature, absolute SHAP values were first averaged across participants within that test fold. Fold-level mean absolute SHAP values were then averaged across the five folds to obtain the final feature-importance estimate. Modality-level SHAP contribution was calculated by summing the mean absolute SHAP values of all features belonging to each modality and normalizing this sum by the total SHAP contribution across all modalities. This procedure ensured that feature importance reflected contributions observed across held-out samples rather than a single training fold.

Strict leakage control was applied throughout the pipeline. First, task-evoked GLM regions were identified using training subjects only within each fold. Second, feature standardization and any feature transformation were fitted on the training data only. Third, the fitted transformations were applied to the test fold without using test labels or distributional information. Fourth, classifiers were trained only on the training fold and evaluated once on the corresponding test fold (Varoquaux, 2018; Scheinost et al., 2019; Poldrack et al., 2020; Eklund et al., 2016; Nichols and Holmes, 2002; Pedregosa et al., 2011; Abraham et al., 2014).

This leakage-controlled design was particularly important because neuroimaging datasets often contain high-dimensional features and limited sample sizes. Without fold-specific preprocessing, test information can inadvertently enter feature selection or normalization, leading to overoptimistic performance estimates (Varoquaux, 2018; Scheinost et al., 2019; Poldrack et al., 2020; Eklund et al., 2016; Nichols and Holmes, 2002). In addition, the relatively large fold-level variability observed in some performance metrics likely reflects both the clinical heterogeneity of adolescent BPD and the sensitivity of multimodal predictive models to participant composition across cross-validation folds. Such variability is not uncommon in moderate-sized psychiatric neuroimaging datasets, particularly when strict leakage-controlled validation procedures are applied.

### 2.12 Evaluation metrics

The primary performance metric was AUC, because it evaluates discriminative ranking ability independently of a fixed decision threshold and is therefore appropriate for comparing classifiers across feature settings. Secondary metrics included accuracy (ACC), F1 score, precision, recall/sensitivity, and specificity. These secondary metrics were reported to characterize clinically relevant trade-offs between false-positive and false-negative predictions. Predicted probabilities were converted into binary predictions using a threshold of 0.5, which was chosen as the default decision boundary for probabilistic binary classifiers and corresponds to equal prior probability. We note that alternative thresholds may be preferable for specific clinical screening or confirmatory applications (Poldrack et al., 2020; Eklund et al., 2016; Nichols and Holmes, 2002).

For each classifier and feature setting, metrics were computed in each fold and then summarized as mean and standard deviation across the five folds. Fold-level predictions and summary metrics were saved for downstream visualization and statistical comparison.

### 2.13 Reproducibility and implementation

All machine learning experiments were implemented in Python using scikit-learn-compatible classifiers. A fixed random seed of 42 was used throughout the analysis. The same five stratified cross-validation folds were used across all feature settings and classifiers to ensure fair comparison. For stochastic procedures, including random sampling, PCA where applicable, and classifier training when relevant, random states were fixed within each fold. All preprocessing operations, including scaling, feature selection, PCA, GLM-derived region definition, and classifier fitting, were performed using the training fold only and then applied to the corresponding held-out test fold. The complete experimental pipeline generated fold-level metrics, prediction probabilities, classifier-wise summaries, modality comparison tables, and interpretation outputs for downstream manuscript reporting (Pedregosa et al., 2011; Abraham et al., 2014).

### 2.14 Reviewer-requested sensitivity and behavioral analyses

To evaluate demographic confounding, age and sex alone were first assessed using five-fold out-of-fold logistic regression. An exact age-by-sex matched subset was then formed without replacement (89 BPD and 89 HC participants), and existing held-out random-forest predictions from the primary cross-validation were restricted to this subset. Stratified bootstrap resampling was used to estimate 95% confidence intervals. This analysis tests the robustness of held-out predictions after demographic balancing but does not retrain region definition or model fitting within the matched subset. Behavioral performance was compared between groups for happy and neutral Go omission-error percentages, No-Go commission-error percentages, and Go reaction times using Welch two-sample tests, with Benjamini-Hochberg correction across six comparisons and Hedges g as the effect-size estimate.

**Table 1:** Feature settings evaluated in the machine learning experiments.

Feature setting	Included modalities	Interpretation
FC	Functional connectivity	Single imaging connectivity modality
GLM	Task-evoked GLM-derived temporal features	Single task-activation modality
Clinical	Clinical variables	Single phenotypic modality
FC + GLM	FC and GLM	Imaging-only multimodal setting
FC + Clinical	FC and clinical variables	Connectivity plus phenotype
GLM + Clinical	GLM and clinical variables	Task activation plus phenotype
All	FC, GLM, and clinical variables	Full multimodal setting

**Table 2:** Classifiers and main hyperparameter settings.

Classifier	Model family	Main settings
LR	Linear classifier	L2 penalty; C = 0.5; balanced class weights; maximum iterations = 5,000
SVM	Kernel-based classifier	RBF kernel; C = 0.7; gamma = scale; probability output enabled; balanced class weights
KNN	Instance-based classifier	k = 7; distance weighting; Euclidean distance
GaussianNB	Probabilistic classifier	Gaussian likelihood assumption
MLP	Neural network classifier	Hidden layers = 32 and 16; ReLU activation; Adam optimizer; early stopping
RF	Tree ensemble	300 trees; maximum depth = 4; minimum leaf samples = 5; square-root feature sampling; balanced class weights
XGBoost/GradientBoosting	Boosting ensemble	150 estimators; maximum depth = 2; learning rate = 0.03; regularized boosting configuration

**Table 3:** Best-performing classifier within each feature setting based on AUC.

Feature setting	Best classifier	ACC	AUC	F1	Precision	Recall	Specificity
FC	LR	0.621 ± 0.043	0.664 ± 0.053	0.626 ± 0.063	0.600 ± 0.044	0.662 ± 0.113	0.582 ± 0.087
Clinical	RF	0.689 ± 0.082	0.776 ± 0.067	0.671 ± 0.108	0.698 ± 0.120	0.672 ± 0.174	0.707 ± 0.181
GLM	RF	0.800 ± 0.064	0.881 ± 0.055	0.760 ± 0.089	0.891 ± 0.034	0.671 ± 0.136	0.922 ± 0.027
FC + GLM	RF	0.812 ± 0.068	0.878 ± 0.053	0.767 ± 0.092	0.944 ± 0.060	0.655 ± 0.136	0.961 ± 0.039
FC + Clinical	GaussianNB	0.653 ± 0.066	0.734 ± 0.046	0.573 ± 0.114	0.697 ± 0.050	0.498 ± 0.159	0.798 ± 0.052
GLM + Clinical	RF	0.800 ± 0.061	0.881 ± 0.054	0.758 ± 0.084	0.905 ± 0.060	0.663 ± 0.135	0.930 ± 0.042
All	RF	0.816 ± 0.068	0.883 ± 0.048	0.770 ± 0.092	0.955 ± 0.052	0.655 ± 0.136	0.969 ± 0.033

**Table 4:** Classification performance of different classifiers using the full multimodal feature set.

Classifier	ACC	AUC	F1	Precision	Recall	Specificity
LR	0.717 ± 0.064	0.821 ± 0.064	0.693 ± 0.077	0.733 ± 0.086	0.670 ± 0.142	0.759 ± 0.098
SVM	0.760 ± 0.120	0.820 ± 0.097	0.738 ± 0.111	0.807 ± 0.143	0.695 ± 0.150	0.819 ± 0.175
KNN	0.820 ± 0.055	0.847 ± 0.078	0.790 ± 0.071	0.904 ± 0.072	0.712 ± 0.126	0.922 ± 0.061
GaussianNB	0.816 ± 0.088	0.771 ± 0.107	0.775 ± 0.106	0.942 ± 0.096	0.663 ± 0.128	0.960 ± 0.069
MLP	0.788 ± 0.097	0.799 ± 0.114	0.757 ± 0.102	0.870 ± 0.136	0.687 ± 0.143	0.882 ± 0.142
**RF**	**0.816 ± 0.068**	**0.883 ± 0.048**	**0.770 ± 0.092**	**0.955 ± 0.052**	**0.655 ± 0.136**	**0.969 ± 0.033**
XGBoost	0.757 ± 0.069	0.824 ± 0.055	0.719 ± 0.068	0.851 ± 0.145	0.639 ± 0.103	0.867 ± 0.152

Bold values indicate the performance of the best-performing classifier selected on the basis of AUC.

## 3 Results

Following the workflow shown in Figure 1, all feature settings and classifiers were evaluated under the same five-fold cross-validation protocol. The results are reported from three perspectives: overall classification performance across feature settings, comparison of classifiers under the full multimodal setting, and modality-level contribution analysis.

**Figure 1:**
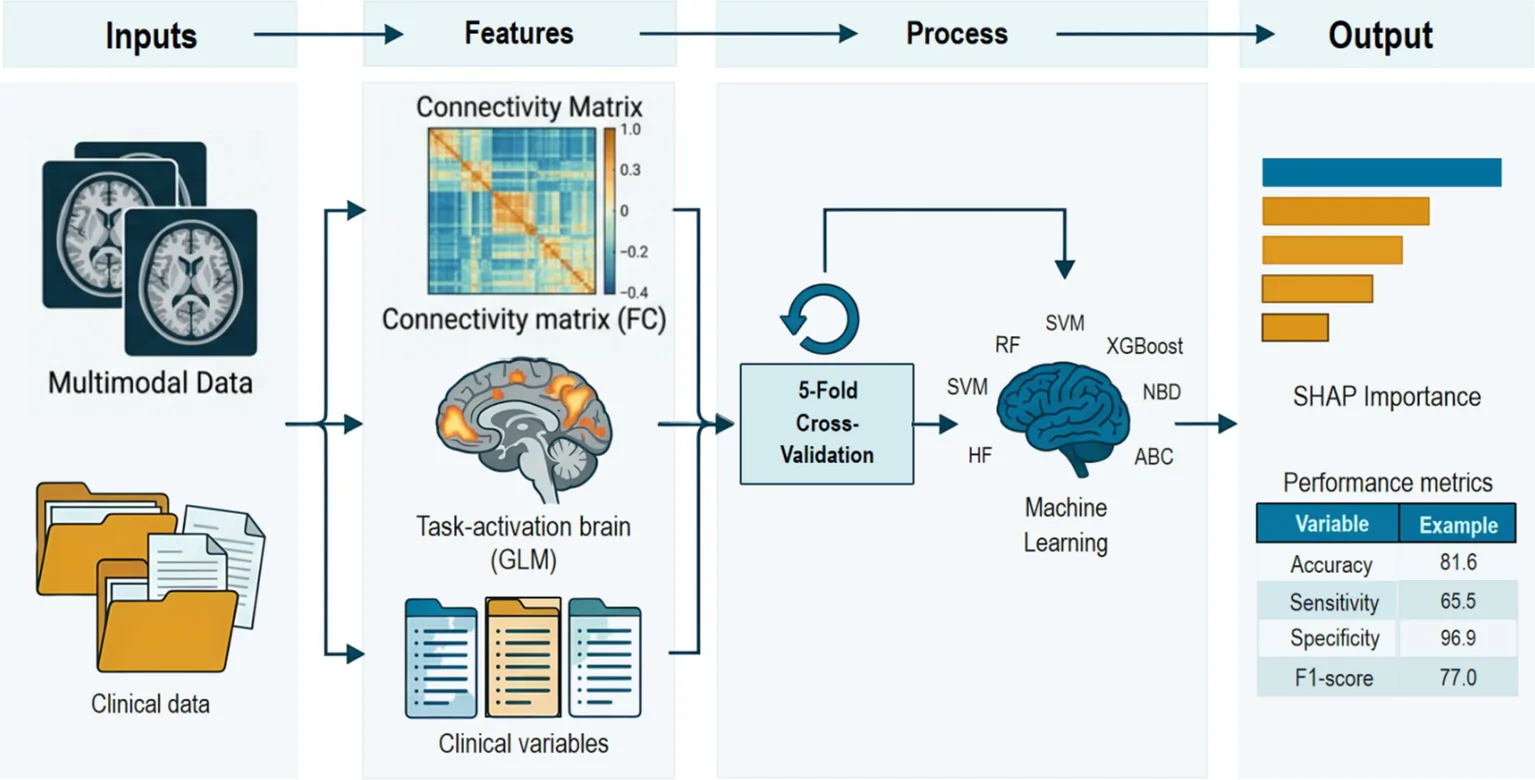
Overall workflow of the multimodal machine learning framework for individualized adolescent BPD classification. The framework consists of four main stages: input, feature construction, machine learning process, and output. Multimodal inputs include task-fMRI data and clinical data. Three feature domains are constructed, including functional connectivity matrices, task-evoked GLM-derived brain activation features, and clinical variables. These features are evaluated under five-fold cross-validation using multiple machine learning classifiers. The final outputs include classification performance metrics, such as accuracy, sensitivity, specificity, and F1 score, as well as SHAP-based feature importance for model interpretation. Icons reproduced with permission.

### 3.1 Demographic and clinical characteristics

The BPD group was slightly younger than the HC group [15.52 ± 1.47 vs. 15.93 ± 0.89 years; *t*(249) = −2.65, *p* = 0.008]. Sex distribution differed significantly between groups (chi-square = 14.40, *p* = 0.0001): the BPD group had a higher proportion of females (68.0%) compared to the HC group (43.4%). Years of education did not differ significantly between groups [9.80 ± 1.62 vs. 9.64 ± 0.84; *t*(249) = 0.99, *p* = 0.323].

#### 3.1.1 Demographic sensitivity analysis

Age and sex alone yielded a five-fold out-of-fold AUC of 0.643. Exact matching produced 89 pairs with identical mean age (15.96 years) and female proportion (61.8%) in both groups. In this matched subset, existing held-out predictions yielded an AUC of 0.866 (bootstrap 95% CI, 0.812–0.916) for GLM-RF and 0.869 (95% CI, 0.815–0.920) for All-RF. These results reduce, but do not eliminate, concern that demographic imbalance explains classification because the models and fold-specific regions were not retrained within the matched subset.

#### 3.1.2 Behavioral performance

After Benjamini-Hochberg correction, the BPD group showed lower neutral Go omission-error percentages (3.998% ± 2.495% vs. 5.255% ± 2.928%; Welch *t* = −3.667, *p* = 0.00030, *q* = 0.00180, Hedges *g* = −0.460) and lower neutral No-Go commission-error percentages (2.681% ± 1.552% vs. 3.194% ± 1.726%; *t* = −2.478, *p* = 0.01386, *q* = 0.04159, *g* = −0.311). Happy Go omission-error percentages, happy No-Go commission-error percentages, and happy or neutral Go reaction times did not differ significantly after correction. Thus, the BPD group did not show a generalized behavioral deficit across task conditions.

### 3.2 Overall classification performance

The classification results demonstrated that task-evoked GLM-derived features provided the most informative single-modality representation for distinguishing adolescents with BPD from healthy controls. Across all feature settings and classifiers, the highest AUC was achieved by All-RF, with an AUC of 0.883 ± 0.048 and an accuracy of 0.816 ± 0.068. The highest accuracy and F1 score were obtained by GLM + Clinical-KNN, with an accuracy of 0.824 ± 0.060 and an F1 score of 0.790 ± 0.075. These results indicate that the full multimodal representation achieved the strongest discriminative ranking ability, whereas the GLM plus clinical setting provided a favorable balance between accuracy and F1 score (Krause-Utz et al., 2014; Schulze et al., 2016; Ruocco et al., 2013; Ruocco et al., 2012; Degasperi et al., 2021; Silbersweig et al., 2007; Minzenberg et al., 2007; Herpertz et al., 2001; Donegan et al., 2003; van Zutphen et al., 2015; Hare et al., 2008; Tottenham et al., 2011; Brown et al., 2015; Orrù et al., 2012; Arbabshirani et al., 2017; Vieira et al., 2017; Woo et al., 2017; Varoquaux, 2018; Scheinost et al., 2019; Poldrack et al., 2020; Eklund et al., 2016; Nichols and Holmes, 2002).

Critically, however, the improvement of the full multimodal model over the GLM-only models was modest, indicating that most predictive information was captured by task-evoked neural dynamics in this dataset. This classification pattern is consistent with HAPI, but it is correlational and is not specific to predictive processing; conventional accounts based on visual attention, salience allocation, and executive inhibitory control provide plausible alternative explanations.

As shown in Figure 2, models incorporating GLM-derived task-evoked features consistently outperformed FC-only and clinical-only models. The full multimodal model achieved the highest AUC, while the GLM-only model showed highly comparable performance, suggesting that task-evoked GLM-derived features represented the dominant discriminative feature source (Tables 3) (Krause-Utz et al., 2014; Schulze et al., 2016; Ruocco et al., 2013; Ruocco et al., 2012; Degasperi et al., 2021; Silbersweig et al., 2007; Minzenberg et al., 2007; Herpertz et al., 2001; Donegan et al., 2003; van Zutphen et al., 2015; Hare et al., 2008; Tottenham et al., 2011; Brown et al., 2015).

**Figure 2:**
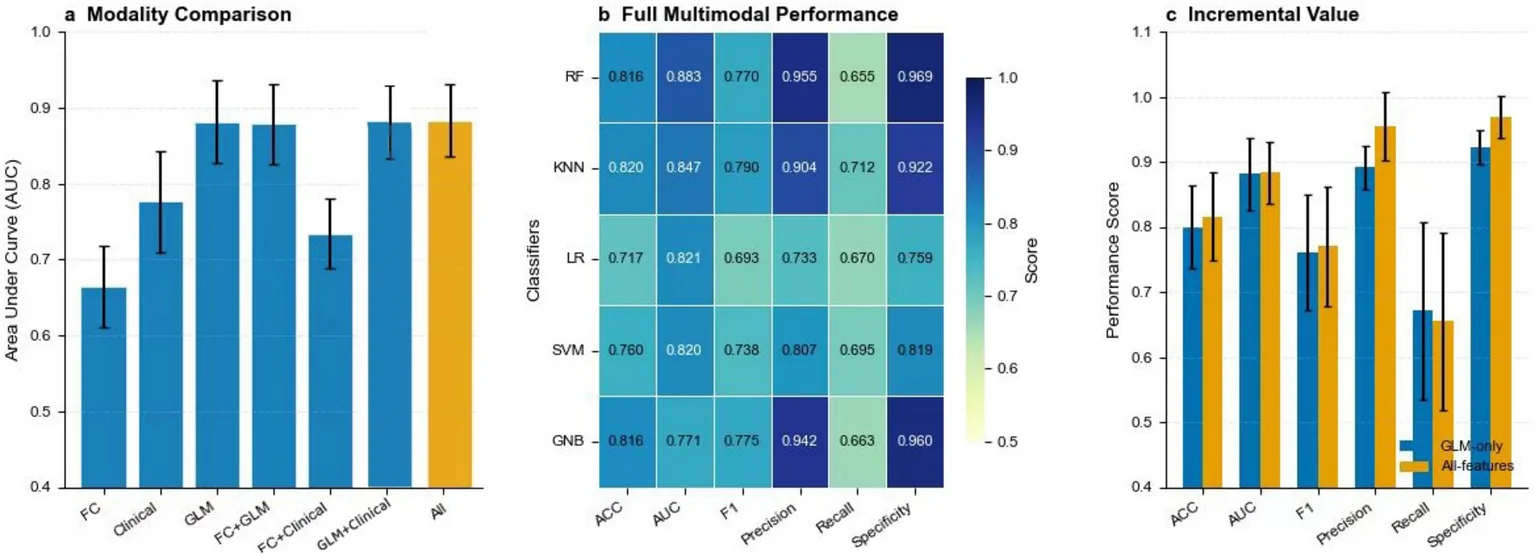
Classification performance overview across feature settings and classifiers. **(a)** Best AUC values obtained under the displayed feature settings, including FC, Clinical, GLM, FC + GLM, FC + Clinical, and the full multimodal feature setting. The GLM + Clinical result is reported in Table 3 and was omitted from this visual summary to avoid redundancy because its AUC was nearly identical to the GLM-only result. Error bars indicate standard deviations across five-fold cross-validation. **(b)** Heatmap of classification performance under the full multimodal feature setting across representative machine learning classifiers. The displayed metrics include ACC, AUC, F1 score, precision, recall/sensitivity, and specificity. Each cell shows the mean score across five folds. **(c)** Performance comparison between the GLM-only RF model and the full multimodal RF model. The full multimodal model achieved slightly higher AUC, precision, and specificity, whereas the GLM-only model showed comparable overall performance, suggesting that task-evoked GLM-derived features contributed the dominant discriminative information.

### 3.3 Comparison of full multimodal classifiers

Under the full multimodal feature setting, the random forest classifier achieved the highest AUC, indicating strong ranking performance for separating BPD and HC participants (Tables 4). KNN achieved the highest accuracy among full-feature models and showed the highest recall, whereas GaussianNB and RF produced high precision and specificity. This pattern reflects a relatively conservative classification profile in which false-positive predictions are reduced at the cost of lower sensitivity and the potential omission of some true BPD cases. The clinical desirability of this trade-off depends on the intended translational application. In screening contexts, higher sensitivity may be prioritized to minimize missed cases, whereas in confirmatory or decision-support settings, higher specificity and precision may be preferable, particularly in clinically heterogeneous adolescent populations. At a more applied level, different classifiers emphasized distinct diagnostic trade-offs, with ensemble models such as random forest favoring higher specificity, and instance-based models such as KNN achieving a more balanced sensitivity–specificity profile. However, these variations did not substantially alter the overall pattern of results, further reinforcing the robustness of the task-evoked predictive signal across modeling assumptions (Breiman, 2001; Cortes and Vapnik, 1995; Cover and Hart, 1967; Friedman, 2001; Chen and Guestrin, 2016; Lundberg and Lee, 2017).

### 3.4 Modality contribution analysis

The modality-level comparison further highlighted the importance of GLM-derived task-evoked features. The best FC-only model achieved an AUC of 0.664 ± 0.053, and the best clinical-only model achieved an AUC of 0.776 ± 0.067. By contrast, the best GLM-only model achieved an AUC of 0.881 ± 0.055, approaching the full multimodal result of 0.883 ± 0.048. This pattern indicates that most of the predictive signal was already captured by task-evoked neural dynamics, with FC and clinical variables providing only modest complementary contributions (Biswal et al., 1995; Rubinov and Sporns, 2010; Smith, 2012; Power et al., 2012; Satterthwaite et al., 2012; Tzourio-Mazoyer et al., 2002; Woo et al., 2017).

The combination of GLM and clinical features also produced competitive performance, achieving an AUC similar to the GLM-only and full multimodal models. However, FC plus clinical features did not reach the same level of performance, reinforcing the observation that task-evoked GLM-derived information was essential for robust classification. Overall, the classification results support the use of task-based fMRI-derived GLM features as a clinically relevant and machine learning-compatible representation for individualized BPD identification.

To further interpret the contribution of different feature sources and brain regions, SHAP analysis was performed for the RF classifiers using both the GLM-only and full multimodal feature settings. SHAP values were computed independently within each cross-validation fold using predictions generated for held-out test participants only. For each feature, absolute SHAP values were averaged across test samples within the corresponding fold, and fold-specific mean absolute SHAP values were subsequently averaged across the five folds to obtain a stable cross-validated estimate of feature importance. For modality-level analyses, SHAP values were summed across features belonging to the same modality and normalized relative to the total SHAP contribution across modalities. As shown in Figure 3a, the full multimodal RF model was predominantly driven by task-evoked GLM-derived features, which accounted for 83.1% of the total SHAP contribution. Functional connectivity and clinical features contributed 9.5 and 7.5%, respectively. This result is consistent with the classification analysis, where the GLM-only RF model achieved performance close to that of the full multimodal RF model. Therefore, the SHAP results further support the conclusion that task-evoked GLM-derived temporal features were the dominant source of discriminative information in this cohort (Lundberg and Lee, 2017). Figure 3b compares the predictive performance of GLM-RF and All-RF. The full multimodal model showed slightly higher AUC and precision than the GLM-only model, indicating that FC and clinical information provided complementary information to improve the confidence and specificity of classification. However, the difference between GLM-RF and All-RF was modest, again suggesting that most of the predictive signal was already captured by the task-evoked GLM feature block. In contrast, recall did not improve substantially after adding FC and clinical features, implying that the full model tended to favor more conservative classification decisions.

**Figure 3:**
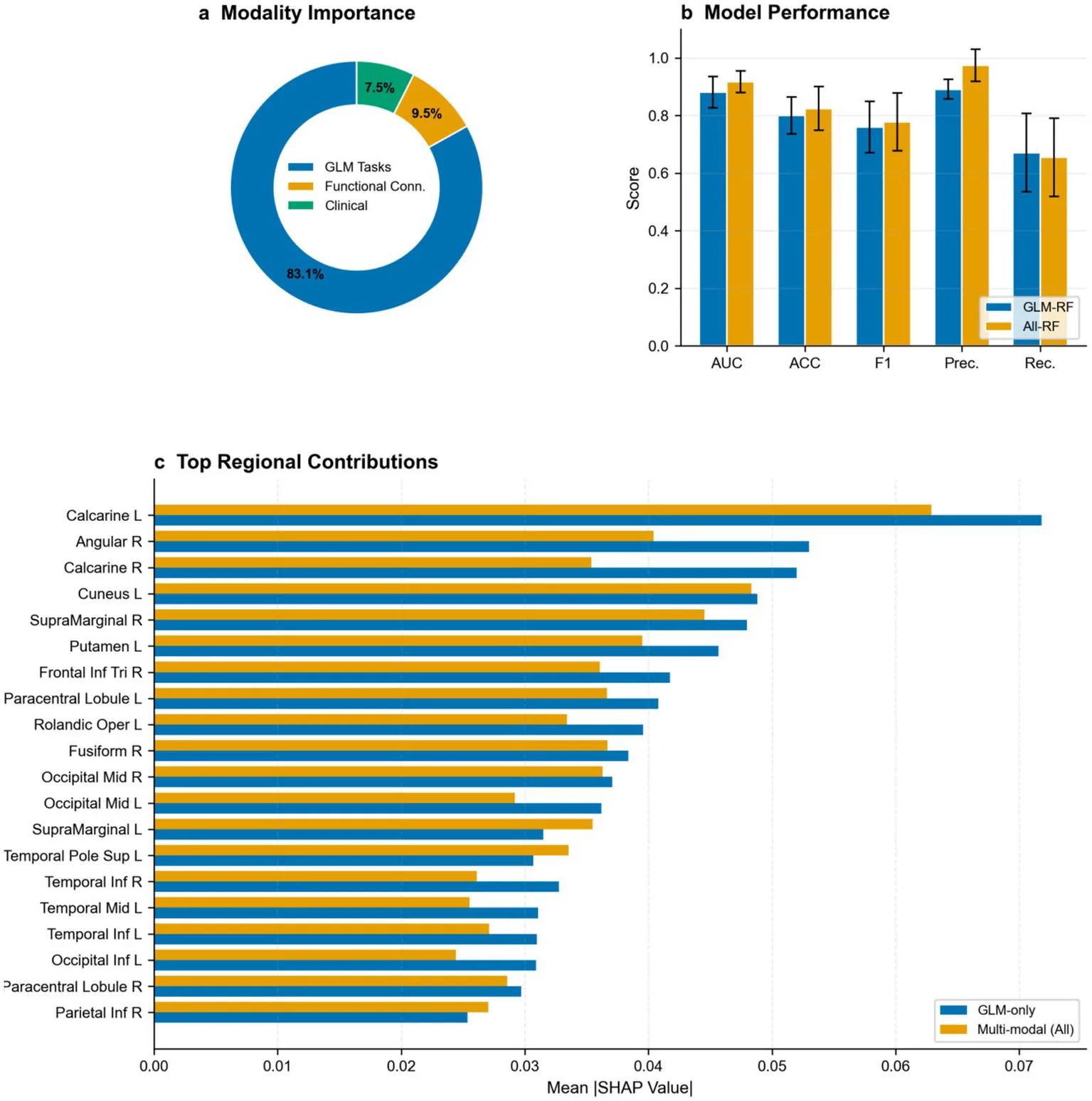
SHAP-based interpretation of the RF classification models. **(a)** SHAP-based modality contribution in the full multimodal RF model. Task-evoked GLM-derived features accounted for the largest proportion of model attribution, whereas FC and clinical features provided smaller complementary contributions. **(b)** Cross-validation performance comparison between GLM-RF and All-RF. Error bars indicate standard deviations across folds. **(c)** Top GLM-derived regional contributions ranked by mean absolute SHAP values. Blue bars indicate the GLM-only RF model, and orange bars indicate the full multimodal RF model. The similar regional contribution patterns suggest that the full multimodal model largely preserved the task-evoked GLM-based discriminative signature.

At the neural level, the most influential regions identified by SHAP analysis were distributed across visual/perceptual regions (calcarine cortex, cuneus, occipital cortex), temporoparietal/default-mode hubs (angular and supramarginal gyri), frontal control regions (inferior frontal gyrus), and subcortical structures (putamen). This distributed pattern suggests a hierarchical organization spanning perceptual processing, contextual/self-referential integration, and top-down inhibitory control systems. From a theoretical perspective, these findings are consistent with the hierarchical affective predictive imbalance (HAPI) model, in which dysfunction arises from impaired coordination across multiple levels of a predictive control hierarchy. Specifically, the prominence of perceptual and temporoparietal regions suggests altered processing and contextual integration of emotionally salient stimuli, while the involvement of frontal and subcortical systems reflects disruptions in inhibitory control and response selection. The fact that these patterns emerge most strongly in task-evoked features further supports the interpretation that BPD-related dysfunction is primarily expressed during active affective predictive regulation, rather than in intrinsic network organization alone (see Figures 3, 4).

**Figure 4:**
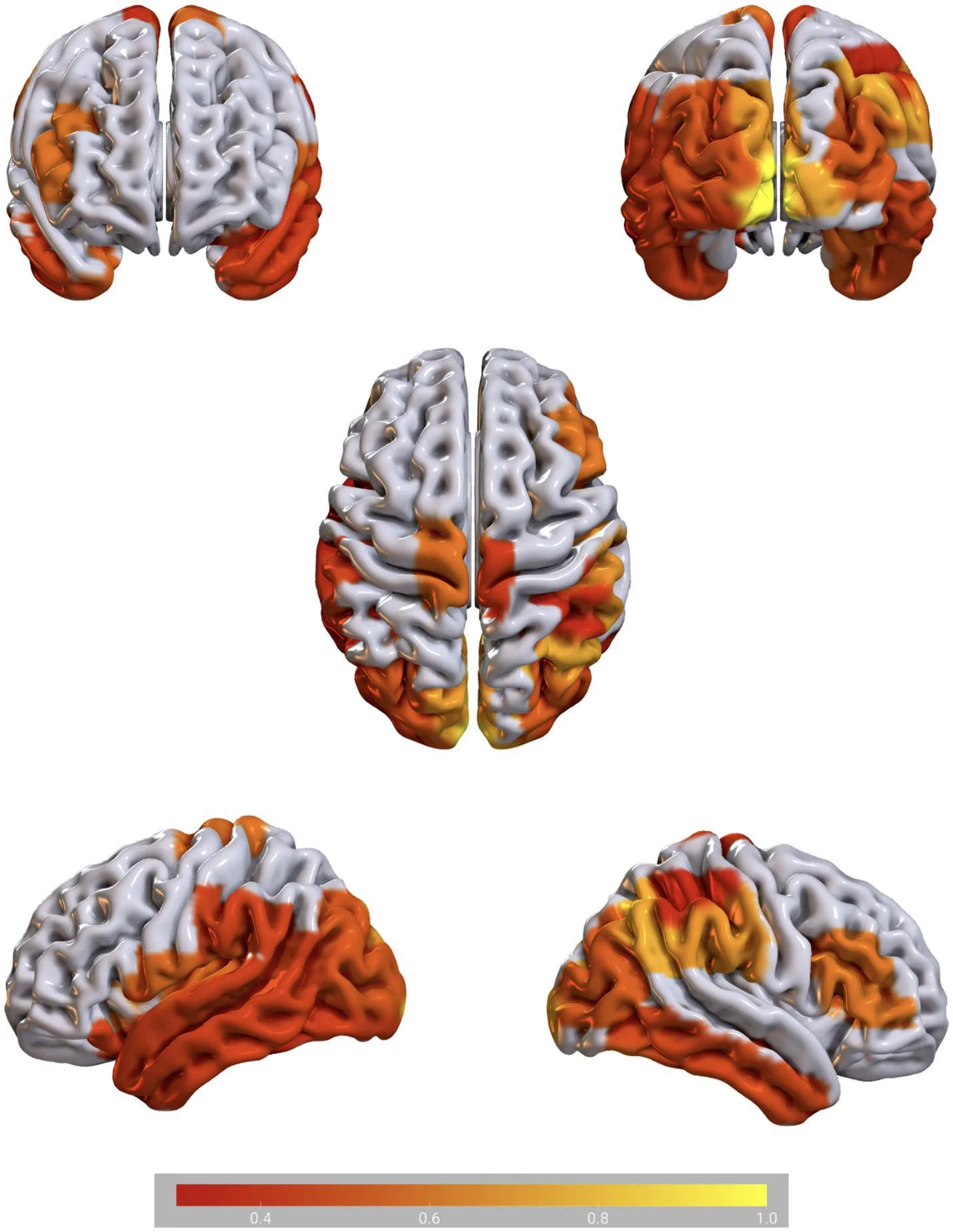
Task-evoked regional contributions (GLM-only) mapped onto the AAL atlas. Regions contributing most to classification are shown with normalized intensities (0–1) based on GLM importance values. The pattern spans occipital (calcarine, cuneus), temporoparietal (angular, supramarginal), frontal (inferior frontal gyrus), and subcortical (putamen) areas. This distributed organization is consistent with hierarchical processing across perceptual, integrative, and control systems, and can be interpreted within the HAPI framework.

Importantly, this pattern is consistent with the HAPI framework. The dominance of GLM-derived task-evoked features indicates that adolescent BPD-related dysfunction is not merely reflected in static connectivity architecture or clinical phenotype, but becomes most evident when the brain is required to dynamically regulate predictions and actions under affective interference. The distributed SHAP pattern further suggests a coordinated imbalance across multiple hierarchical levels, including perceptual encoding, contextual/self-referential integration, and top-down inhibitory control (Varoquaux, 2018; Scheinost et al., 2019; Poldrack et al., 2020; Eklund et al., 2016; Nichols and Holmes, 2002; Lundberg and Lee, 2017).

## 4 Discussion

This study suggests that task-evoked neural dynamics during affective inhibitory control constitute a primary source of discriminative information in adolescent BPD. The observed pattern is consistent with hierarchical predictive-processing accounts, including HAPI, but does not directly test the computational operations proposed by those accounts. The contributing visual, temporoparietal, frontal, and subcortical regions can also be interpreted through established models of visual attention, salience processing, and executive inhibitory control.

A major observation of this study was the dominant role of GLM-derived task-evoked features. Compared with FC-only and clinical-only models, GLM-only models achieved substantially higher classification performance. Within the HAPI framework, this pattern suggests that disease-relevant information in adolescent BPD may emerge more strongly during dynamic affective predictive regulation than from intrinsic large-scale architecture alone. The best FC-only model achieved an AUC of 0.664 ± 0.053, whereas the best GLM-only model achieved an AUC of 0.881 ± 0.055, which was close to the full multimodal model with an AUC of 0.883 ± 0.048. This pattern indicates that most of the discriminative signal was already captured by task-evoked neural dynamics, with only limited additional gain from combining modalities. From a translational perspective, the strong performance of GLM-derived features suggests that task-based neural responses constitute a particularly informative and interpretable biomarker. From a theoretical perspective, this result is consistent with the hierarchical affective predictive imbalance (HAPI) model, which posits that dysfunction in adolescent BPD emerges most strongly when neural systems are required to dynamically regulate behavior under emotionally salient conditions. The emotional Go/No-Go paradigm directly engages these processes by requiring the integration of affective stimulus processing with response inhibition, thereby placing demands on predictive control mechanisms. In this context, GLM-derived features capture the moment-to-moment engagement and stability of neural responses, providing a functional readout of how effectively individuals regulate behavior under affective interference (Krause-Utz et al., 2014; Schulze et al., 2016; Ruocco et al., 2013; Ruocco et al., 2012; Degasperi et al., 2021; Silbersweig et al., 2007; Minzenberg et al., 2007; Herpertz et al., 2001; Donegan et al., 2003; van Zutphen et al., 2015; Hare et al., 2008; Tottenham et al., 2011; Brown et al., 2015).

The relatively limited performance of FC-only models suggests that static functional connectivity may not be sufficient to characterize the disorder-relevant information required for individualized BPD classification in this cohort. FC features reflect inter-regional synchronization patterns and can provide useful information about large-scale brain network organization. However, the present results indicate that this intrinsic architecture alone is insufficient to fully characterize disorder-relevant information at the individual level. Instead, dysfunction appears to be more strongly expressed when the system is actively engaged under affective control demands. Compared with task-evoked GLM-derived features, FC measures are less sensitive to transient, context-dependent neural dynamics, which are critical for regulating behavior in emotionally salient situations (Biswal et al., 1995; Rubinov and Sporns, 2010; Smith, 2012; Power et al., 2012; Satterthwaite et al., 2012; Vieira et al., 2017). From the perspective of the hierarchical affective predictive imbalance (HAPI) model, this distinction is expected: FC captures the baseline organization of predictive systems, whereas task-evoked responses reflect their functional deployment during active predictive regulation. The finding that FC + GLM models performed similarly to GLM-only models further suggests that FC provides complementary but non-dominant information, refining but not replacing the core task-evoked signal.

Clinical-only models achieved moderate classification performance, indicating that behavioral and clinical variables contained useful phenotypic information. Variables that directly encoded diagnosis or disease history were excluded to reduce shortcut learning. However, all 38 retained variables were entered without data-driven selection, whereas FC underwent ANOVA filtering and PCA. Consequently, the weaker clinical-only result may partly reflect estimation variance or dimensionality rather than a true absence of clinical signal, and it should not be interpreted as evidence that clinical information lacks predictive value.

The full multimodal model achieved the highest AUC and specificity, suggesting that the integration of FC, GLM-derived, and clinical features can improve the ranking ability and conservative identification capacity of the classifier. However, the gain over the GLM-only model was small. This finding is theoretically informative because it suggests that task-evoked dynamics already captured most of the disease-relevant signal, while FC and clinical variables contributed complementary but secondary information. This pattern indicates that additional FC and clinical features provided complementary information, but most predictive information was already contained in the GLM-derived feature block. The full multimodal RF model showed high precision and specificity, suggesting that it was more conservative in identifying BPD cases and tended to reduce false-positive predictions. In contrast, recall/sensitivity did not substantially improve after adding FC and clinical variables. This diagnostic trade-off highlights the need to report multiple performance metrics rather than relying only on accuracy or AUC. For clinical screening scenarios, sensitivity may be prioritized to reduce missed cases, whereas for confirmatory or decision-support scenarios, specificity and precision may be more important (Varoquaux, 2018; Scheinost et al., 2019; Poldrack et al., 2020; Eklund et al., 2016; Nichols and Holmes, 2002).

The comparison across classifiers further showed that different machine learning algorithms emphasized different aspects of classification performance. Under the full multimodal feature setting, RF achieved the highest AUC and specificity, whereas KNN achieved the highest accuracy, F1 score, and recall/sensitivity. These results indicate that RF may be more suitable when stable ranking performance and high specificity are desired, while KNN may provide a more balanced classification profile when sensitivity and F1 score are emphasized. The variation across classifiers also supports the importance of systematic benchmarking. Instead of relying on a single selected classifier, evaluating multiple model families provides a more complete understanding of how different algorithms interact with multimodal neuroimaging and clinical features (Breiman, 2001; Cortes and Vapnik, 1995; Cover and Hart, 1967; Friedman, 2001; Chen and Guestrin, 2016; Lundberg and Lee, 2017). More broadly, the observed performance differences are consistent with the HAPI interpretation that task-evoked affective regulation may be a prominent level at which adolescent BPD-related information becomes behaviorally and neurally expressed; however, this pattern is not specific to predictive-processing mechanisms.

The SHAP analysis linked classification performance to a distributed neural pattern, showing that the predictive signal was not localized to a single region but involved multiple levels of a hierarchical system. At the modality level, the full multimodal RF model was predominantly driven by GLM-derived features, which accounted for the largest proportion of model attribution. FC and clinical features contributed smaller proportions, consistent with the performance comparison showing that GLM-only models were already close to the full multimodal model. At the regional level, the most influential features were distributed across visual/perceptual regions (calcarine cortex, cuneus, occipital cortex), temporoparietal/default-mode hubs (angular and supramarginal gyri, temporal regions), frontal control regions (inferior frontal gyrus), and subcortical structures (putamen). These regions broadly overlap with systems undergoing protracted adolescent maturation, including sensory integration and inhibitory-control networks. Recent developmental and genomics-informed work has emphasized that psychiatric vulnerability during adolescence may reflect interactions between brain maturation, neurodevelopmental risk, and cell-specific molecular profiles. From this perspective, the present task-evoked fMRI findings provide a functional-level pattern that may complement, rather than replace, structural and molecular accounts of adolescent psychopathology. Future multimodal work integrating task-fMRI, structural trajectories, and transcriptomic or molecular annotations may help clarify how HAPI-related predictive features relate to broader neurodevelopmental mechanisms (Krause-Utz et al., 2014; Schulze et al., 2016; Ruocco et al., 2013; Ruocco et al., 2012; Degasperi et al., 2021; Silbersweig et al., 2007; Minzenberg et al., 2007; Herpertz et al., 2001; Donegan et al., 2003; van Zutphen et al., 2015; Lundberg and Lee, 2017; Wu et al., 2025; Kuang et al., 2023; Ran et al., 2026).

However, the SHAP-derived regions should be interpreted as predictive features rather than causal biomarkers. Model attribution methods identify features that contribute to classification decisions, but they do not establish whether these regions are mechanistically responsible for BPD symptoms (Lundberg and Lee, 2017). In addition, because GLM-derived regions were selected within each training fold, some high-contribution regions may appear only in specific folds. Therefore, stable regions repeatedly observed across folds should be emphasized in the main interpretation, while fold-specific high-contribution regions should be treated as exploratory findings. This distinction is particularly important in psychiatric neuroimaging, where sample size, heterogeneity, and feature dimensionality can influence apparent regional importance (Varoquaux, 2018; Scheinost et al., 2019; Poldrack et al., 2020; Eklund et al., 2016; Nichols and Holmes, 2002; Lundberg and Lee, 2017). Importantly, several SHAP-relevant cortical and subcortical regions identified in the present study overlap with systems undergoing substantial maturation during adolescence, including frontostriatal, salience, and temporoparietal networks involved in affective regulation, sensory integration, and inhibitory control. From a broader neurodevelopmental perspective, future studies integrating task-fMRI-derived biomarkers with transcriptomic or molecular datasets may help clarify the biological pathways underlying these disease-relevant neural states, including mechanisms related to synaptic maturation, cortical specialization, and experience-dependent neurodevelopmental plasticity. These interpretations should remain speculative, however, because the present study did not directly assess molecular or developmental biological markers.

A key methodological strength of this study is the leakage-controlled cross-validation design. In neuroimaging classification, information leakage may occur when feature selection, normalization, dimensionality reduction, or region definition is performed using the full dataset before cross-validation. Such leakage can lead to overly optimistic performance estimates. To reduce this risk, task-evoked GLM regions were identified using only the training subjects within each fold and then applied to the corresponding held-out test subjects. Similarly, feature standardization and model fitting were performed strictly within the training folds. This design improves the credibility of the reported performance and provides a more realistic estimate of within-sample generalization to unseen participants, although independent external validation remains necessary to establish cross-site translational robustness (Varoquaux, 2018; Scheinost et al., 2019; Poldrack et al., 2020; Eklund et al., 2016; Nichols and Holmes, 2002; Pedregosa et al., 2011; Abraham et al., 2014).

## 5 Limitations and conclusion

Several limitations should be acknowledged. First, although five-fold cross-validation was conducted under strict leakage control, external validation remains necessary. Second, the prespecified hyperparameters reduced optimization bias but may underestimate tuning-sensitive algorithms, including XGBoost; definitive comparisons require fully nested optimization. Third, demographic, behavioral, medication-related, and acquisition-related confounding cannot be completely excluded. The age- and sex-matched sensitivity analysis reduced concern about demographic imbalance, but it evaluated existing held-out predictions rather than retraining the entire feature-definition pipeline in the matched subset. The parent clinical cohort included psychotropic-medication exposure in 22.5% of BPD participants; because medication-stratified task-fMRI analyses were not available for the present revision, residual medication effects on task-evoked signals cannot be excluded.

Fourth, the present study focused on conventional machine learning classifiers. This choice improved interpretability and enabled systematic benchmarking across model families with different inductive biases; deep learning or graph-based approaches may be useful when substantially larger datasets become available.

Fifth, SHAP-derived regional importance should be interpreted as predictive attribution rather than causal or mechanistically specific evidence. Model attribution identifies features that contribute to classification decisions but does not establish whether those regions are responsible for BPD symptoms.

Sixth, the study did not perform a dedicated descriptor-level ablation of the individual temporal statistics used in the GLM-derived feature block. Future work should determine which aspects of response amplitude, variability, first-difference dynamics, and distributional percentiles contribute most robustly to classification performance and generalizability (Varoquaux, 2018; Scheinost et al., 2019; Poldrack et al., 2020; Eklund et al., 2016; Nichols and Holmes, 2002; Lundberg and Lee, 2017).

In summary, this study demonstrates that task-evoked GLM-derived temporal features provide strong discriminative information for individualized classification of adolescent BPD. The findings are consistent with hierarchical predictive-processing accounts and suggest that disorder-related differences are most clearly expressed during task-evoked affective control in this cohort. They also indicate that task-based fMRI combined with leakage-controlled machine learning offers a promising and interpretable approach for capturing disorder-relevant brain states at the individual level, while underscoring the need for independent external validation (Hare et al., 2008; Tottenham et al., 2011; Brown et al., 2015; Woo et al., 2017; Varoquaux, 2018; Scheinost et al., 2019; Poldrack et al., 2020; Eklund et al., 2016; Nichols and Holmes, 2002).

## Data Availability

The datasets presented in this article are not publicly available because the datasets generated and analyzed during the current study are subject to ethical restrictions, institutional regulations, and protections for sensitive human neuroimaging and clinical data from adolescent participants. Requests for access should be directed to yixiaoping@cqu.edu.cn and will be considered subject to applicable ethical and institutional approvals.

## Footnotes

1
http://www.fil.ion.ucl.ac.uk/spm
